# The distribution of breast density in women aged 18 years and older

**DOI:** 10.1007/s10549-024-07269-y

**Published:** 2024-03-18

**Authors:** Dilukshi Perera, Sarah Pirikahu, Jane Walter, Gemma Cadby, Ellie Darcey, Rachel Lloyd, Martha Hickey, Christobel Saunders, Michael Hackmann, David D. Sampson, John Shepherd, Lothar Lilge, Jennifer Stone

**Affiliations:** 1 Genetic Epidemiology Group, School of Population and Global Health, The University of Western Australia, 35 Stirling Highway M431, Perth, WA 6009 Australia; 2 University Health Network, Toronto, ON Canada; 3 Department of Obstetrics and Gynaecology, University of Melbourne and the Royal Women’s Hospital, Melbourne, VIC Australia; 4 Department of Surgery, Royal Melbourne Hospital, The University of Melbourne, Melbourne, VIC, Australia; 5 School of Human Sciences, The University of Western Australia, Perth, WA Australia; 6 Optical and Biomedical Engineering Laboratory School of Electrical, Electronic and Computer Engineering, The University of Western Australia, Perth, WA, Australia; 7 Surry Biophotonics, Advanced Technology Institute and School of Biosciences and Medicine, The University of Surrey, Guildford, Surrey, UK; 8 Epidemiology and Population Sciences in the Pacific Program, University of Hawaii Cancer Center, Honolulu, HI USA; 9 Medical Biophysics, University of Toronto, Toronto, ON Canada

**Keywords:** Breast density, Breast cancer, Risk factor, Measurement

## Abstract

**Purpose:**

Age and body mass index (BMI) are critical considerations when assessing individual breast cancer risk, particularly for women with dense breasts. However, age- and BMI-standardized estimates of breast density are not available for screen-aged women, and little is known about the distribution of breast density in women aged < 40. This cross-sectional study uses three different modalities: optical breast spectroscopy (OBS), dual-energy X-ray absorptiometry (DXA), and mammography, to describe the distributions of breast density across categories of age and BMI.

**Methods:**

Breast density measures were estimated for 1,961 Australian women aged 18–97 years using OBS (%water and %water + %collagen). Of these, 935 women had DXA measures (percent and absolute fibroglandular dense volume, %FGV and FGV, respectively) and 354 had conventional mammographic measures (percent and absolute dense area). The distributions for each breast density measure were described across categories of age and BMI.

**Results:**

The mean age was 38 years (standard deviation = 15). Median breast density measures decreased with age and BMI for all three modalities, except for DXA-FGV, which increased with BMI and decreased after age 30. The variation in breast density measures was largest for younger women and decreased with increasing age and BMI.

**Conclusion:**

This unique study describes the distribution of breast density measures for women aged 18–97 using alternative and conventional modalities of measurement. While this study is the largest of its kind, larger sample sizes are needed to provide clinically useful age-standardized measures to identify women with high breast density for their age or BMI.

**Supplementary Information:**

The online version contains supplementary material available at 10.1007/s10549-024-07269-y.

## Introduction

Mammographic breast density is the radiographic white appearance of fibroglandular breast tissue seen on a mammogram and is one of the strongest and most prevalent breast cancer risk factors [1]. High breast density increases breast cancer risk [2, 3] and reduces the sensitivity of mammography, as cancers also appear white on a mammogram, resulting in a "masking" effect [4–6]. The risk of cancer going undetected at screening (i.e., interval cancer) is primarily attributed to increased breast density in asymptomatic women [7, 8].

Other than being female, age is the strongest predictor of breast cancer risk and is mainly responsible for the large variation of breast density across women [1]. Breast density decreases with age but breast cancer risk increases with age. Therefore, the true risk factor is breast density *for a woman's age*. However, it is currently difficult to determine whether a woman has high breast density for her age in a clinical setting as there is no age-standardized measure. Knowing a woman’s breast density percentile for her age, for example, could help identify those who may benefit from risk-reducing strategies and/or younger women (< 40 years of age) who may benefit from early breast screening.

BMI also negatively confounds the association between breast density and breast cancer risk. Higher BMI increases the risk of breast cancer in postmenopausal women but is negatively associated with percent breast density [9]. Increasing BMI is associated with increasing total breast size [9]; therefore, measures of percent breast density are often negatively associated with BMI, given how it is calculated. The association between BMI and absolute measures of breast density is typically negative but it has been shown to be positively associated when examining volumetric- instead of area-based measures [10–12].

Sprague and colleagues estimated the proportion of screen-aged women with heterogeneously dense or extremely dense breasts by categories of age and BMI [1]. While these prevalence estimates are useful, they are not helpful for individual comparison of breast density for a given age or BMI, nor do they extend to women aged < 40 years who are not recommended for routine mammographic screening. As it has been shown that reducing breast density through endocrine therapy can reduce breast cancer risk [13], risk-reducing strategies targeting younger women could provide the largest benefits [14]. A continuous measure of breast density, instead of categorical measures, could help monitor changes in breast density and be used as a biomarker of the effectiveness of risk-reducing interventions. Overall, a safe and simple standardized approach for routine assessment and reporting of breast density for women of all ages may be useful for both women and health professionals to determine whether a woman has high breast density for their age and/or BMI.

There are several alternative methods of measuring breast density that are safe for women of all ages and also correlate closely with mammographic measures, including magnetic resonance imaging (MRI) [15, 16], dual X-ray absorptiometry (DXA) [17–21] and optical breast spectroscopy (OBS) [22–26]. MRI measures breast tissue water content to estimate breast density but unfortunately is not an efficient modality for routine breast density assessment due to accessibility issues, cost and invasiveness. DXA measures fibroglandular breast tissue volume and is relatively more accessible and less costly/invasive but still requires small doses of ionizing radiation and therefore is not suitable for potentially pregnant women. OBS is a cost-effective, safe, non-invasive, non-ionizing method that uses the spectral response of light transmission to analyze volumetric breast tissue composition without imaging and suitable for use in younger women [25–27]. The OBS approach measures optical properties from light scattering and absorption and assesses levels of breast tissue chromophores, including water and collagen [23, 27]. Similar optical spectroscopy studies have shown higher collagen concentrations in dense breasts [28]; hence, incorporating breast collagen and water content measurements could be more meaningful when directly estimating breast density [29].

This study illustrates the distribution of breast density in Australian women aged 18–97 across categories of age and BMI using three different modalities: OBS, DXA and mammography. Age- and BMI-categorized distributions enable individual comparison of breast density to other women of similar age or BMI which in future could be used to inform individual breast cancer risk assessment.

## Methods

### Study participants and recruitment

Six groups of women were sourced from two different studies: the Breast Density Pilot Study and The Raine Study [30]. The Pilot Study participants (Pilot Study 1, *n* = 539) were women aged between 18 and 40 recruited via the University of Western Australia's Crowd Research website [31], Register4 website [32], and word of mouth from March 2016 to June 2018. These women were recalled 2+ years after their first OBS scan (Pilot Study 2, *n* = 283). The Raine Study is an ongoing prospective pregnancy cohort study established in 1989 with various prospective follow-ups [33, 34]. Female participants from the Raine Study were recruited at age 27 (Gen2-27, *n* = 452) and recalled again at age 28 years (Gen2-28, *n* = 356). The overlap between Gen2-27 and Gen2-28 is 217. The biological mothers (Gen1, *n* = 460) and grandmothers (Gen0, *n* = 104) of Raine Study participants were also recruited as part of the 28-year-old follow-up (when combined, referred to as Gen0/1; *n* = 564). This included biological mothers and grandmothers of all female participants as well as mothers and surviving maternal grandmothers of male participants.

Study participation included completion of an extensive epidemiological questionnaire, measurement of height and weight, an OBS scan, and both a whole-body and breast-DXA scan. Gen2-27 participants were not invited to have a DXA scan. Gen0 and Gen1 participants consented for a copy of existing mammograms to be released from the population-based screening program, BreastScreen Western Australia.

### Exclusion criteria

Women who were treated for breast cancer (endocrine-, radio- or chemo-therapy) or had breast surgery affecting both breasts (e.g., mastectomy, lumpectomy, augmentation, and/or reduction) were not eligible to participate. Women who had breast surgery (e.g., benign breast lump removed) on one breast were included and breast density measures from the contralateral breast were used. Women aged < 18 and > 40 were not eligible to participate in Pilot Study 1. Women who were pregnant or trying to get pregnant were not eligible for a DXA scan due to low-dose radiation exposure.

### Measuring OBS breast density

The design and technical details of the OBS device have been published previously [22, 23, 27]. Briefly, the OBS device consists of one of four breast cups (sized to approximately match bra cups A, B, C, and D) and the main controller board. Each cup comprises two light-emitting modules containing 13 lasers spanning the 660–1050 nm spectral range [27] (Supplementary Fig. 1A) and six sensitive photodetectors that capture the fractional wavelength-dependent light transmission from the two light-emitting modules (Supplementary Fig. 1B). Per breast, the light transmission between the light modules and photodetectors was quantified in up to 12 overlapping breast volumes.

For the OBS scan, women were asked to remove their clothing from the waist up and change into a front-opening hospital gown (Supplementary Fig. 1A). Before starting the scan, a reference measurement of a silicon phantom standard was performed. Participants were then positioned sitting upright, holding the device securely over one breast at a time. During the scan, real-time quality control checks were performed by the research assistant and, when required, measurements were repeated. Women participating in Pilot Study 2 were asked to have an extra left breast scan to assess reliability. A second reference measurement of the silicon phantom was completed at the end of the breast scans for each participant.

### OBS data processing

As described previously [22, 23, 26, 27], the resulting OBS output is processed to produce four main chromophore concentrations representing breast tissue volume composition: water, collagen, haemoglobin, and lipids. Herein, the light attenuation percentage of the water (%water) refers to breast density. We also investigated the combination of %water and %collagen as a measure of breast density, as %collagen is a component of dense breast tissue.

Repeated OBS measures of the left breasts were assessed and the Pearson correlation coefficients for %water and %lipids were 0.46 and 0.71, respectively. Differences in OBS-%water in the left breasts greater than 10% were examined manually and the closest measurement to the right breast was selected for inclusion (*n* = 23). Otherwise, the first measurement of the left breast was used. The correlation of %water between the left breasts was 0.78 after exclusion of the 23 outlying measures. The mean of the chosen left and right breasts was used to calculate the final measures for each participant.

### Measuring DXA breast density

All DXA breast density measurements were completed by a trained research assistant following the standard operating procedures described previously [17–19, 26] and briefly summarized here. Women were required to remove all jewellery and their clothing from the waist up and change into a front-opening hospital gown. During the scan, the participant lay on their side (lateral decubitus position) exposing each breast scanned one at a time. All participants were asked to have an extra left breast scan to assess reliability.

A MATLAB (MathWorks, Natick, MA) program (the University of Hawaii, version 5) was used to compute percent fibroglandular volume (%FGV) and absolute fibroglandular volume (FGV) (cm^3^) from the DXA breast images [19]. A single trained reader (DP) measured all the DXA images.

Repeated DXA measures of the left breasts were assessed, and differences in %FGV greater than 10% or differences in FGV greater than 200cm^3^ were inspected manually for quality control. Images with artifacts were excluded. Otherwise, the first measurement of the left breast was used. Repeated measurements were completed for a random sample of 149 images to assess intra-rater reliability and generated very similar results (intra-rater intra-class correlation coefficients > 0.9).

### Measuring mammographic density

A single observer (JSt) measured the cranio-caudal views of the most recent full-field digital mammograms for Gen0/1 participants, obtained from BreastScreen Western Australia. The Cumulus software program was used (Sunnybrook Health Sciences Centre, Toronto, Canada), producing measures of percent dense area (PDA) (%) and absolute dense area (DA) (cm^2^). A random selection of 10% of image measurements was repeated to assess the interclass correlation, which was > 0.9 for both measures.

### Statistical methods

Descriptive statistics were used to summarize participant characteristics and breast density measurements using R software (version 4.2.0). Rain cloud plots were created for each breast density measure by ten-year age categories and three BMI categories.

## Results

**Fig. 1:**
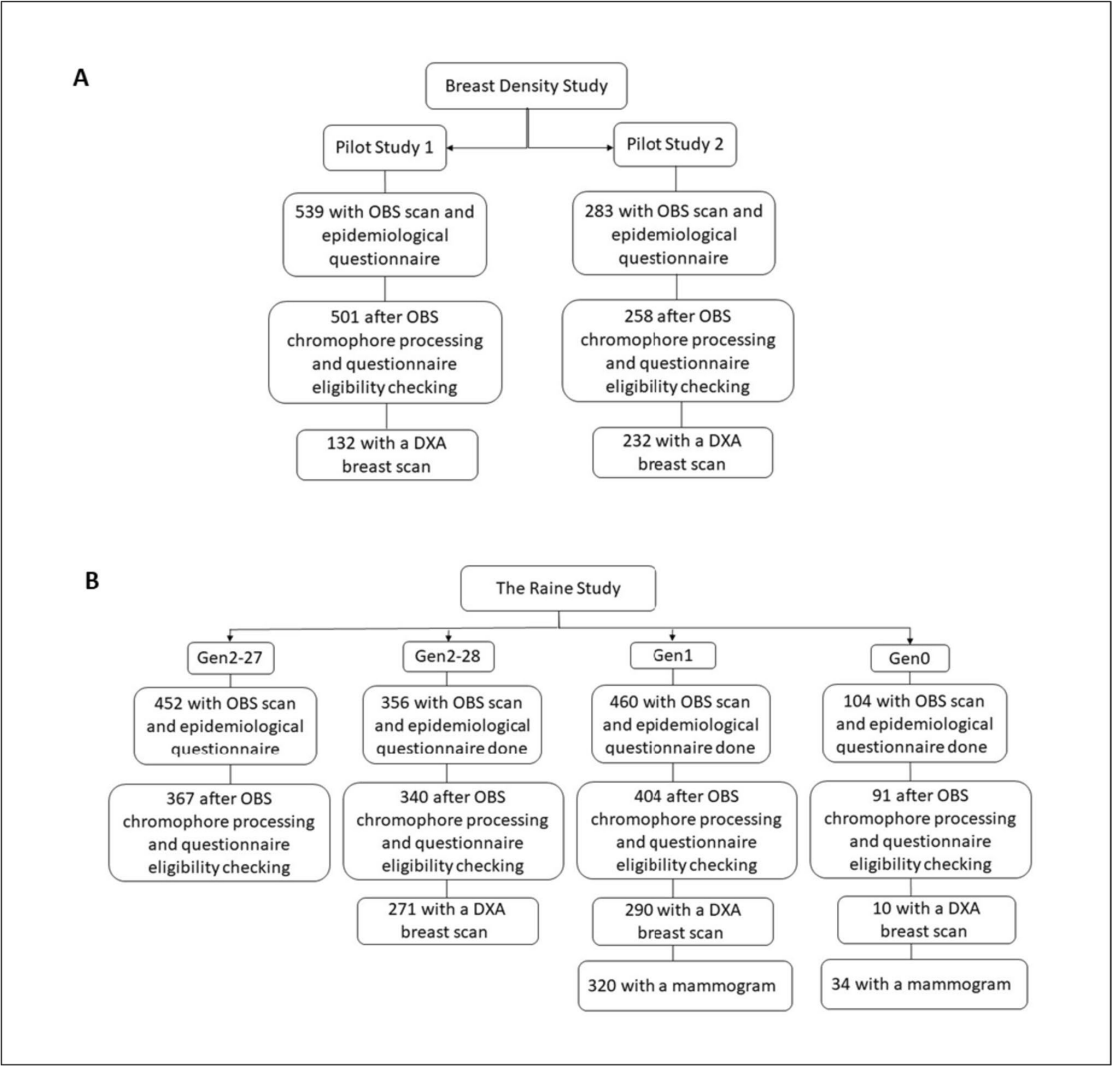
Flowchart describing the number of participants in each study group. **a**; The Breast Density Pilot Study had two groups—Pilot Study 1 and Pilot Study 2. Both groups completed an OBS scan and questionnaire, and a subset of participants also had a DXA breast scan. **b**; The Raine Study had four groups of participants. Female participants from the Raine Study were recruited at age 27 (Gen2-27) and again at age 28 years (Gen2-28). Their biological mothers (Gen1) and grandmothers (Gen0) were recruited for the 28-year-old follow-up. All four groups had an OBS scan and a questionnaire, while a subset of women had a DXA breast scan and a mammogram. OBS; optical breast spectroscopy. DXA; dual energy X-ray absorptiometry

Figure 1 is a flowchart of women sampled. In total, 2,194 women had an OBS scan and completed the epidemiological questionnaire. After processing the OBS data and checking questionnaire eligibility data, a total of 1,961 women remained eligible for analysis. Of these, a subset of 935 women also had a DXA breast density scan. From Gen0/1, mammograms from 354 women (320 mothers and 34 grandmothers) were also available. The mean time difference between the OBS scan date and mammogram date was approximately 1 year (SD 1.52 years).

Table 1 describes the characteristics of participants stratified by measurement modality. Overall, the age of participants with OBS measures ranged from 18 to 97 years, with a median of 31.6 years (inter-quartile range (IQR) 27.5–45.9 years). The median age for the subset of women with DXA measures was 36 years and (IQR 28.2–54.2 years) and the age range for women who had a mammogram available was 45–88 years, with a median of 59.4 years (IQR 55.6–64.0 years).

**Table 1:** Characteristics of participants, stratified by breast density measuring methods

N	OBS	DXA	Mammograms
	1961	935	354
Age (years)			
Median (IQR)	31.6 (27.5–45.9)	36.0 (28.2–54.2)	59.4 (55.6–64.0)
	N (%)	N (%)	N (%)
18–29	924 (47.1)	361 (38.6)	0
30–39	469 (23.9)	214 (22.9)	0
40–49	106 (5.4)	78 (8.3)	15 (4.2)
50–59	226 (11.5)	165 (17.6)	178 (50.3)
60–69	147 (7.4)	107 (11.4)	130 (36.7)
70 +	89 (4.5)	10 (1.1)	31 (8.8)
BMI (kg/m^2^)			
Median (IQR)	24.6 (21.8–28.7)	24.8 (21.9–28.9)	26.9 (23.9–31.1)
	N (%)	N (%)	N (%)
< 25	1049 (53.5)	482 (51.6)	120 (33.9)
25–30	524 (26.7)	262 (28.0)	130 (36.7)
> 30	388 (19.8)	191 (20.4)	104 (29.4)

BMI body mass index; OBS optical breast spectroscopy; DXA dual X-ray energy attenuation.

The median BMI of all participants with OBS measures was 24.6 kg/m^2^ (IQR 21.8–28.7 kg/m^2^), similar to that of the subset with DXA measures (24.8 kg/m^2^ (IQR 21.9–28.9 kg/m^2^)). The median of BMI was slightly higher in the older subset of women with mammograms (26.9 kg/m^2^ (IQR 23.9–31.1 kg/m^2^)).

**Fig. 2:**
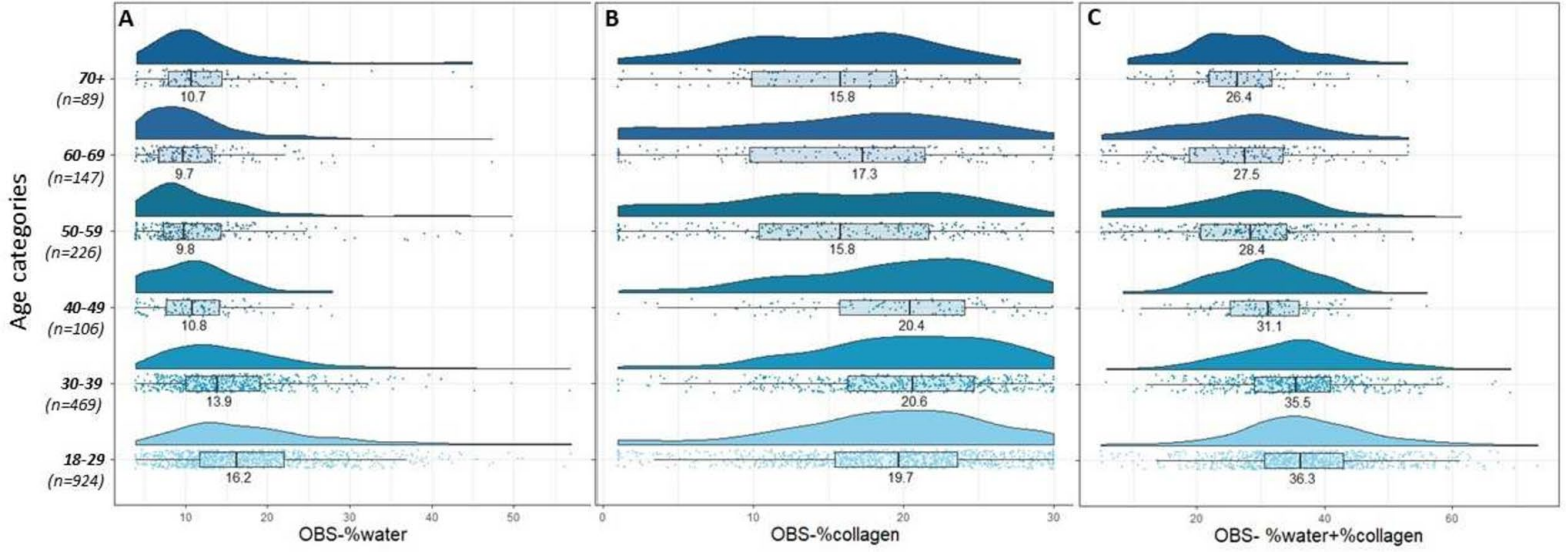
The distribution of OBS measures, stratified by age category. **a**. Distribution of OBS-%water stratified by age categories; **b**. distribution of OBS-%collagen stratified by age categories; **c**. *distribution of OBS-%water* + *%collagen stratified by age categories*. Within each age category all points are plotted, along with a histogram and a boxplot. The number labeled on the histogram is the median of the distribution.

*OBS measures by age:* Raincloud plots for the OBS measures across age categories are shown in Figs. 2A–C. From Fig. 2A, the histograms for OBS-%water were right skewed. Median OBS-%water was highest in the youngest age groups and steadily decreased (from 18–29 to 60–69). Variation of OBS-%water was also highest in the two youngest age groups (18–29 and 30–39) and decreased with age (IQR’s 11.7–22.0 and 10.0–19.0, respectively). In contrast, the distribution of OBS-%collagen in the younger age groups (age < 50) was strongly left skewed with the medians around 20% and post-age 50, the variances were wide with medians around 15–17% (Fig. 2B). For combined OBS-%water + %collagen, the distributions were more symmetrically distributed for all age categories except for 70 + (Fig. 2C). Median %water + %collagen was highest in the two youngest age groups (18–29 and 30–39) and steadily decreased with age (from 40–49 to 70 +).

**Fig. 3:**
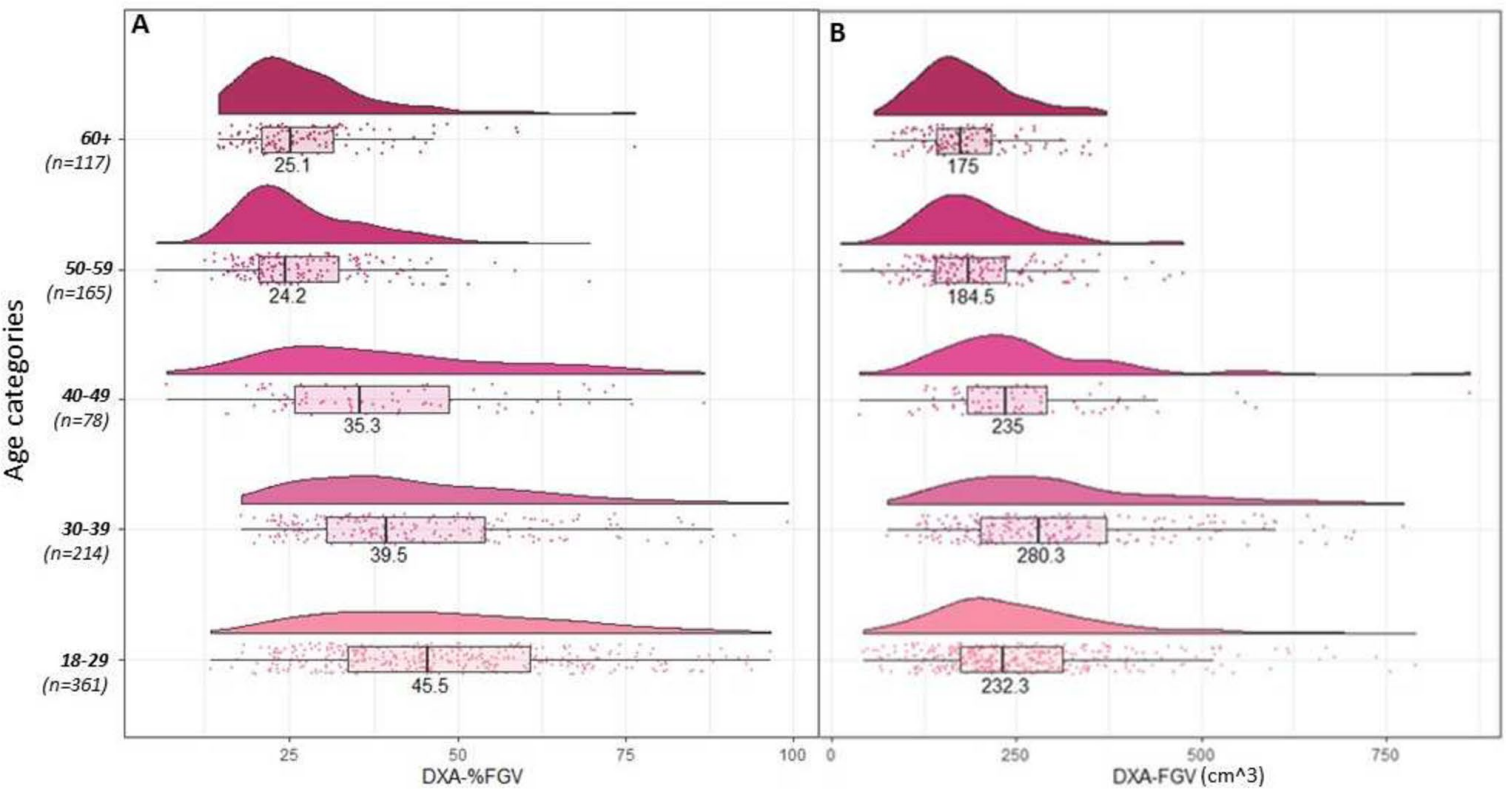
The distribution of DXA breast density measures, stratified by age category. **a**. Distribution of percent fibroglandular volume (%FGV) stratified by age categories; **b**. distribution of absolute fibroglandular volume (FGV) stratified by age categories. Within each age category all points are plotted, along with a histogram and a boxplot. The number labeled on the histogram is the median of the distribution.

*DXA measures by age:* Figs. 3A–B shows the raincloud plots for the DXA breast density measures across age categories. There was considerable variation in %FGV for the younger age groups (< 50). However, the median steadily declined from 45.5% (IQR 33.7–60.8%) to 35.3% (IQR 25.7–48.7) in age groups 18–29 to 40–49. From age 50 + , there was less variation but the distribution was right skewed and truncated on the left, similar to OBS-%water. Similarly, for FGV, the variation was larger for the two younger age groups (18–29 and 30–39; IQRs = 174.2–311.8 and 201.0–372.4, respectively), and the medians steadily declined from age 30, with the highest median recorded for the 30–39 years age category (280.3), and the lowest for the 60 + age category (175.0) (Fig. 3B).

**Fig. 4:**
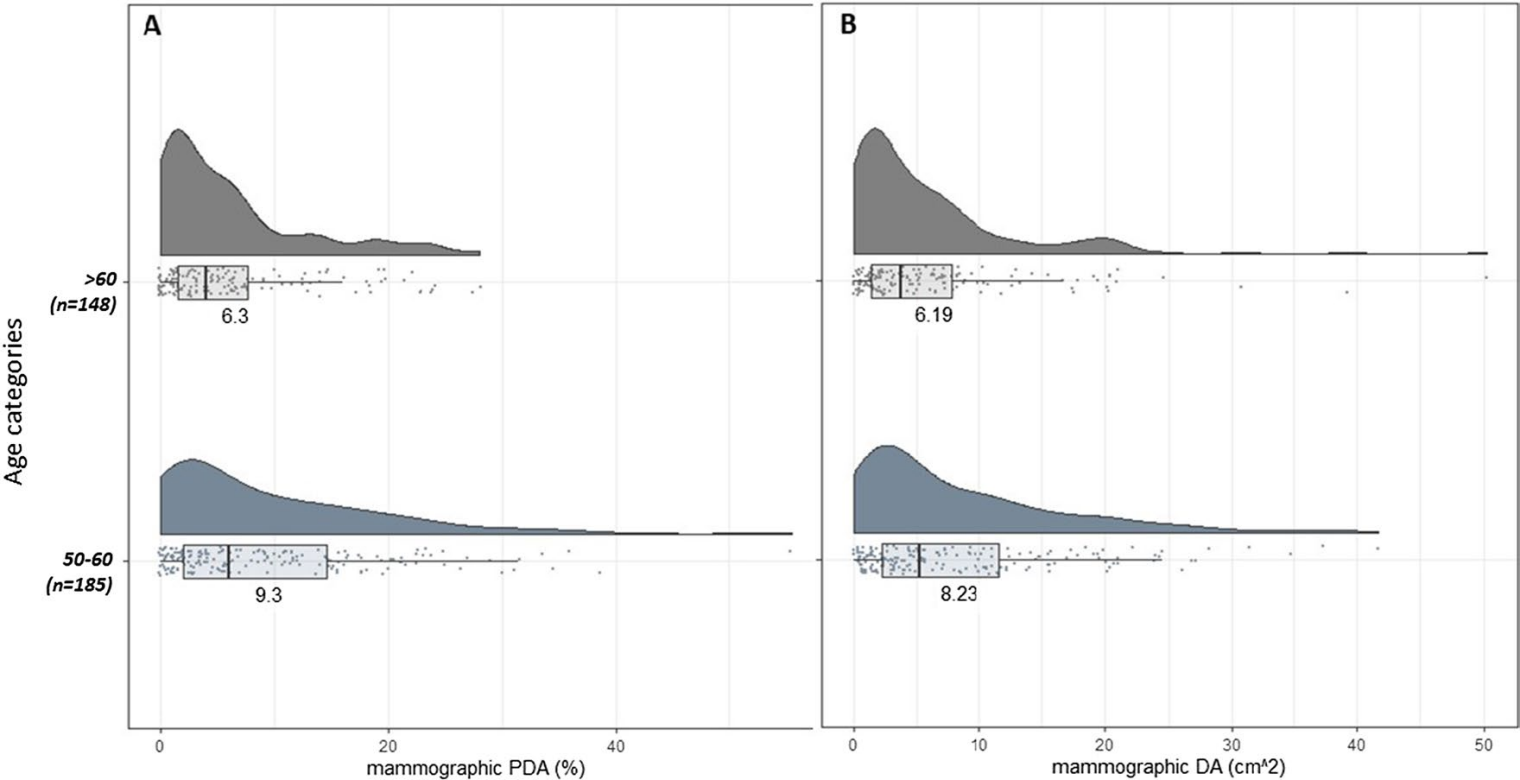
The distribution of mammographic breast density measures, stratified by age category. **a**. Distribution of percent dense area (PDA) stratified by age categories; **b**. distribution dense area (DA) stratified by age categories. Within each age category, all points are plotted, along with a histogram and a boxplot. The number labeled on the histogram is the median of the distribution. * Note- The ages used in these plots were age at mammogram, not age at OBS scan

*Mammographic measures by age (at mammogram):* Fig. 4A–B shows the raincloud plots for the mammographic breast density measures in women over 50. Similar to OBS-%water, the clouds for both PDA and DA were mostly right skewed and the medians decreased with age (from 9.3% to 6.3% for PDA and 8.2 cm^2^ to 6.2 cm^2^ for DA).

**Fig. 5:**
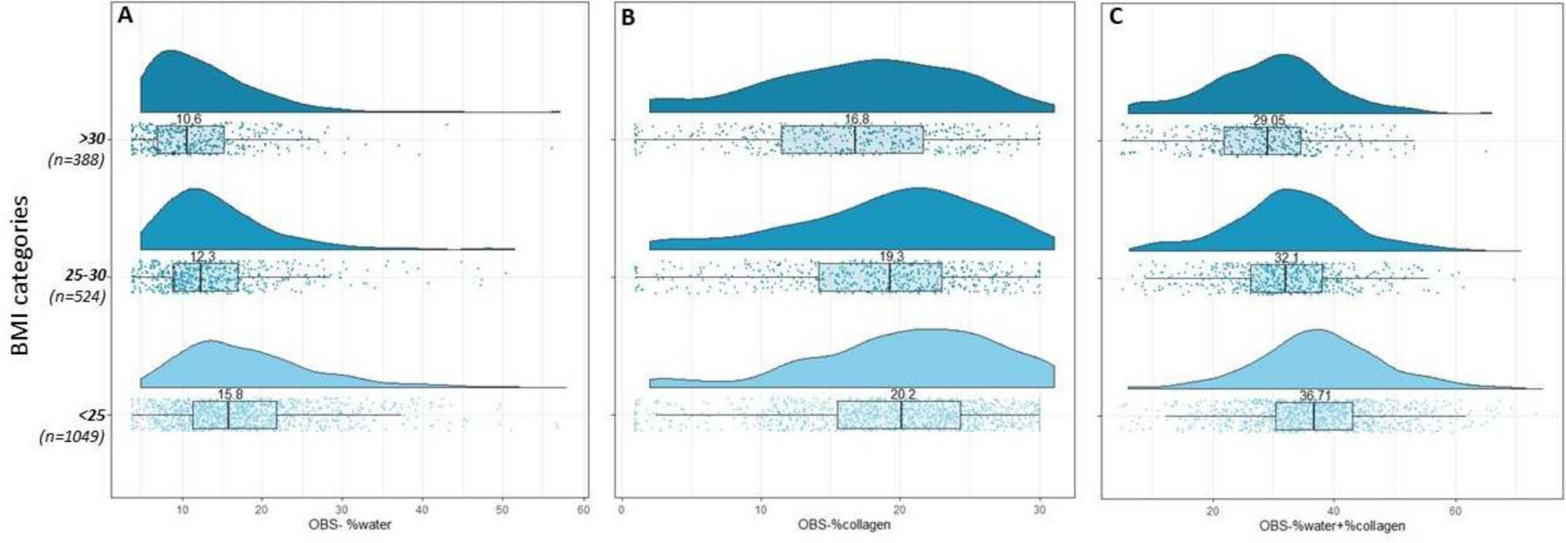
The distribution of OBS measures, stratified by BMI category. Distribution of OBS-%water stratified by BMI categories; **b**. distribution of OBS-%collagen stratified by BMI categories; **c.** distribution of OBS-%water + %collagen stratified by BMI categories. Within each BMI category all points are plotted, along with a histogram and a boxplot. The number labeled on the histogram is the median of the distribution

*OBS measures by BMI*: The median OBS measures were highest in the lowest BMI group and steadily decreased with increasing BMI (Figs. 5A–C). From Fig. 5B, the variation was wide in the distributions of OBS-%collagen across all BMI categories. From Fig. 5C, the distributions of OBS-%water + %collagen combined were largely symmetrically distributed for women with BMI < 25 kg/m^2^.

**Fig. 6:**
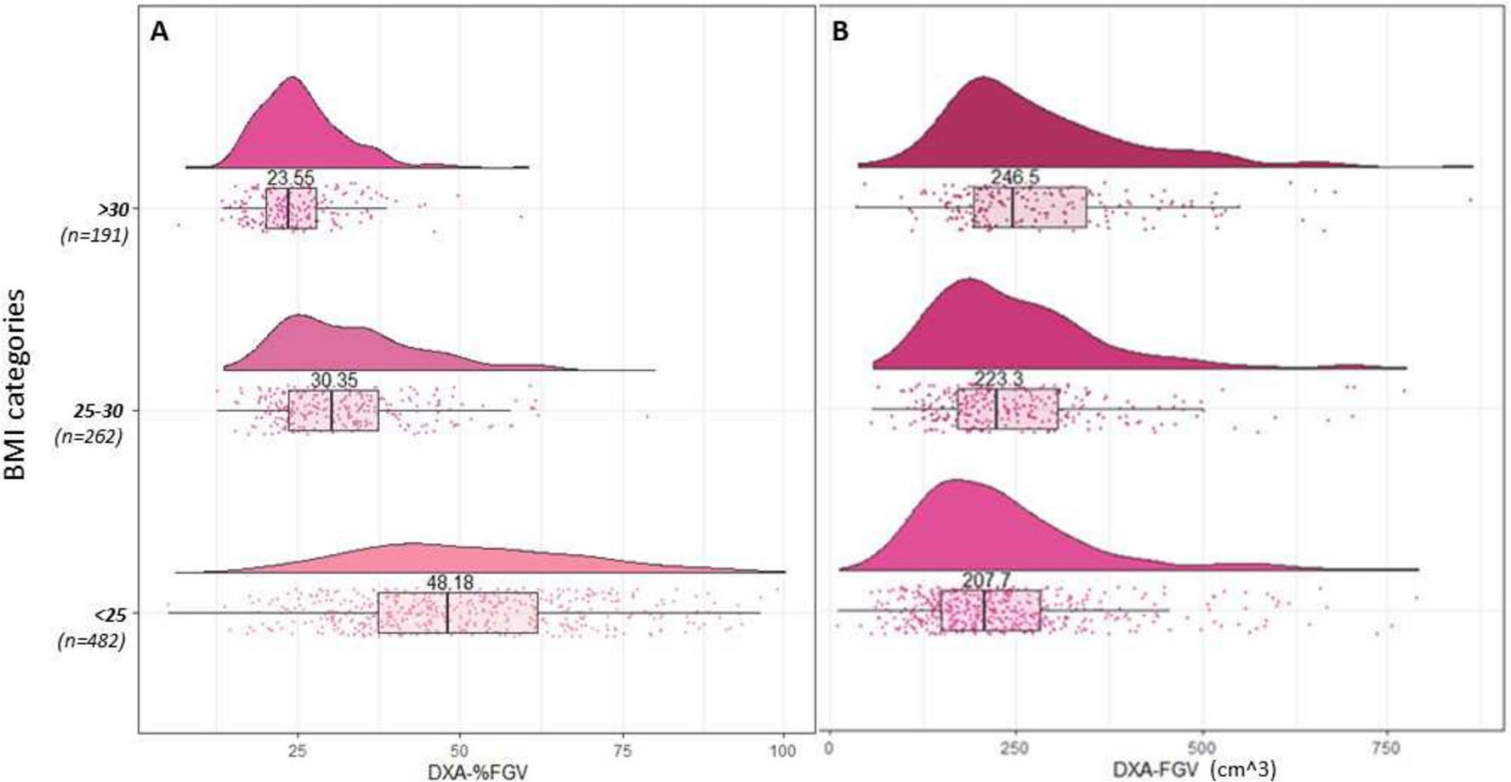
The distribution of DXA breast density measures, stratified by BMI category. **a**. Distribution of percent fibroglandular volume (%FGV) stratified by BMI categories; **b**. Distribution of absolute fibroglandular volume (FGV) stratified by BMI categories. Within each BMI category all points are plotted, along with a histogram and a boxplot. The number labeled on the histogram is the median of the distribution

*DXA measures by BMI*: Fig. 6A–B shows the raincloud plots for the DXA breast density measures across BMI categories. From Fig. 6A, there was larger variation in %FGV in the lowest BMI group, which reduced as BMI increased (IQRs from 37.3–61.9 kg/m^2^ to 20.1–27.8 kg/m^2^). The medians also steadily declined from the lowest to highest BMI category (from 48.2 kg/m^2^ to 23.6 kg/m^2^). In contrast, for FGV, the distributions were largely symmetrical across all BMI categories and the medians steadily increased as BMI increased (Fig. 6B).

**Fig. 7:**
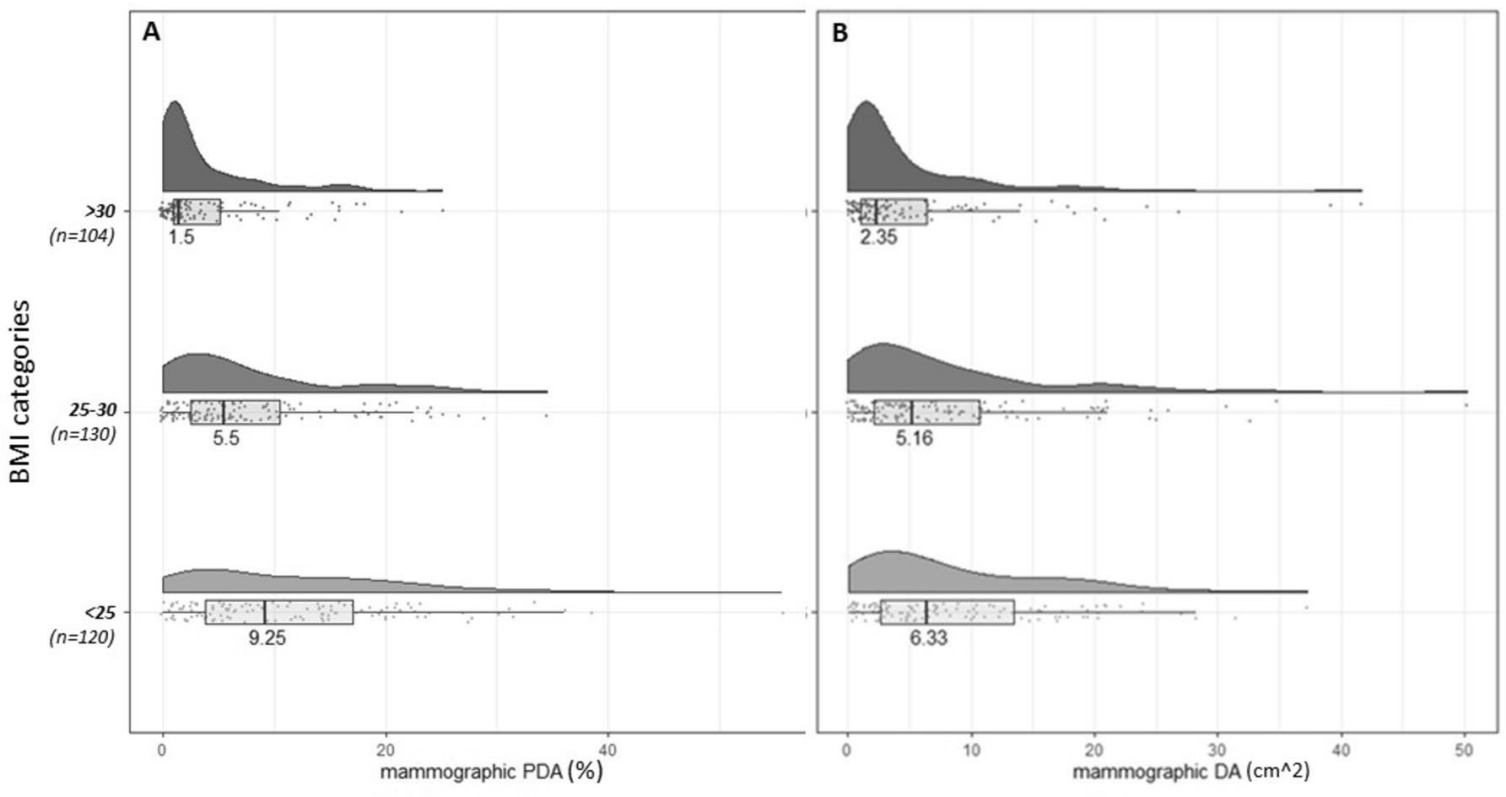
The distribution of mammographic breast density measures, stratified by BMI category. **a**. Distribution of percent dense area (PDA) stratified by BMI categories; **b**. distribution dense area (DA) stratified by BMI categories. Within each BMI category all points are plotted, along with a histogram and a boxplot. The number labeled on the histogram is the median of the distribution

*Mammographic measures by BMI*: The distributions of mammographic PDA and absolute DA across BMI categories are shown in Fig. 7A–B. The distributions for both PDA and absolute DA in all BMI categories were right skewed, with the BMI < 25 kg/m^2^ category having the largest variation. The medians of PDA and DA were highest in the lowest BMI category, and the medians steadily decreased as BMI increased, similar to the OBS measures and DXA-%FGV.

## Discussion

This is the first study to describe the distribution of breast density for women aged 18–97 years measured using three different modalities—OBS, DXA, and mammography. Despite the mix of volumetric and area measures (mammography) estimated from both imaging and non-imaging techniques, we have shown that the median breast density measures decrease with age and BMI for all three modalities. The exception is for DXA-FGV which only decreased after age 30 and increased with increasing BMI. Similarly, the variation in breast density measures was largest for younger women and decreased with increasing age and BMI across all three modalities. We also showed, for the first time, the distribution of non-histologically measured total breast collagen (OBS-%collagen) in these women, a structural tissue component of mammographic breast density [35]. The median OBS-%collagen measures did not decrease linearly with age but decreased rather abruptly after age 50, suggesting a potential menopausal effect.

The negative association between mammographic breast density and age is well established [1]. Consistent with this literature, breast density measured from all three modalities decreased with age. The only exception is perhaps the appearance of a slight increase in the median of OBS-%water in the 70 + age group and of DXA-%FGV in the 60 + group compared to the preceding age category. This is supported by only a modest decrease in the median of mammographic PDA in the 60 + group compared to the 50–60 age group, suggesting that the oldest women in this study (70 +) have slightly higher breast density than one might expect for their age. This is likely a chance finding due to small sample sizes in these age categories. It is also worth noting that all of the women aged 43 and older were mothers and grandmothers (Gen0/1) within the Raine Study and, therefore, all parous women as it is a pregnancy cohort. This potentially explains why their mammographic density measures are relatively low compared to a typical cross-sectional population that would have a mix of both parous and non-parous women. It could therefore also be possible that the rate of decline of breast density with age within parous-only populations is different compared to other sample populations. McCormack and colleagues [36] showed a possible trend between increasing number of live births and rate of change in breast density where nulliparous women experienced a slower rate of decline over time; however, this trend was not statistically significant in a small sample of 645 women. Larger longitudinal studies are needed to confirm the impacts of studying breast density within parous-only sample populations.

The negative trend between age and OBS-%water is also consistent with an MRI study by Boyd and colleagues which also used percent water as an alternative measure for breast density in younger women [15]. They examined three possible models of change in breast density from early adulthood. They concluded that it is most likely that there is large variation in breast density across women at early ages, and the rate of decline with age is potentially greater for those who started with higher breast density [15]. This is consistent with the theory that breast density is established at the age at which the breasts form and is largely determined by genetic factors [37, 38] and then environmental factors act to, on average, decrease breast density as a woman ages [39]. Supplementary Fig. 2 replicates the histograms produced in the MRI study, providing further validation of this theory, with decreasing medians and variation with age for OBS-%water + %collagen and DXA-%FGV.

The trend between median DXA-FGV and age was also negative but only after age 30. Absolute measures of breast density are restricted by total breast size. For example, Maskarinec and colleagues reported higher FGV in mothers than in adolescent daughters [20] who had not reached full breast maturity. Increasing BMI is associated with increasing breast size [9], and in the present study, women in the 18–29 age group had the lowest BMI (Supplementary Fig. 3). Therefore, it could be that FGV within women in the youngest age category (< 30) was limited by breast size.

The negative association between BMI and mammographic breast density is also well established [1]; as body fat increases, the percentage of breast density decreases, largely due to increased breast size [9]. The medians for all three of our percentage breast density measures substantially decreased with clinical categories of BMI, consistent with the literature [1, 9, 40]. The amount of absolute dense area is also thought to decrease with increased BMI; however, the underlying mechanism is not fully understood. Studies investigating absolute dense volume, instead of area, have shown positive associations with BMI [15, 17]. This is consistent with our reported positive trend between median DXA-FGV and BMI.

The three modalities used in this study allowed us to interpret breast density from three different physical manifestations. X-ray based techniques are sensitive to the concentration of atoms with higher z numbers, whereas OBS is sensitive to the concentrations and molecular structure of the chromophores in the interrogated volumes. Using OBS, we reported percent breast density using light attenuation properties of breast tissue volume components–%water and %collagen–while DXA breast density and mammographic breast density provide volumetric- and area-based measures of fibroglandular tissue, respectively. Although the three modalities use different technology and units of measurement, they have all been reported to be highly correlated with each other [17, 21, 24, 41]. This study confirms that the distributions of the percentage-based breast density measures from all three modalities were consistently associated with age and BMI.

The strengths of this study include the application of three breast density measuring modalities in overlapping samples of women aged 18–97 enabling comparison between conventional mammographic measures and two alternative measures, OBS and DXA, which can be used safely in women of all ages. This study enables extrapolation of what is known about the distribution of mammographic breast density in older women to younger populations in whom prevention strategies could be more effective [42–44]. While this study is the largest of its kind, much larger sample sizes are needed to provide clinically useful age-standardized measures to identify women with high breast density for their age or BMI.

This study had some limitations. The processing applied to full-field digital mammograms may be responsible for the relatively low means of both PDA and DA for screen-aged women. Reduced sample size of women with mammograms and/or DXA scans also required us to collapse data across several age categories, preventing direct comparison with the other modalities.

## Conclusion

This study described the distribution of breast density in Australian women aged 18–97 years across categories of age and BMI using alternative and conventional modalities. This new evidence is an important first step toward enabling all adult women to understand their individual breast density compared to other women of similar age and BMI. Although breast cancer risk assessment tools are improving, inclusion of mammographic breast density information is restricting individual risk assessment to screen-aged women and ignores younger women, whose breast density is higher and for whom primary prevention strategies may be more effective [42–44]. As such, future advancement of our findings could provide clinically useful, age-standardized measures of breast density that can be obtained safely and easily to inform breast cancer risk assessment at any age or BMI.

### Supplementary Information

Below is the link to the electronic supplementary material.

Supplementary file1 (DOCX 2102 kb)

## Data Availability

The data that support the findings of this study are available on request from the corresponding author pending approval. The data are not publicly available due to privacy or ethical restrictions.

## Abbreviations

DXA: Dual X-ray absorptiometry
OBS: Optical breast spectroscopy
BMI: Body mass index
%FGV: Percent fibroglandular dense volume
FGV: Absolute fibroglandular dense volume
NFGV: Non-dense fibroglandular volume
OBS-%water: Optical breast spectroscopy percent water
OBS-%lipid: Optical breast spectroscopy percent lipid
OBS-%collagen: Optical breast spectroscopy percent collagen
OBS-%water + collagen: Optical breast spectroscopy percent water and percent collagen
