# Supplementary material for: The distribution of breast density in women aged 18 years and older
Source: Breast Cancer Res Treat. 2024 Mar 18;205(3):521–31. doi: 10.1007/s10549-024-07269-y (PMC11101556; doi:10.1007/s10549-024-07269-y)

**Title: The distribution of breast density in women aged 18+**

Authors: Dilukshi Perera^1^; Sarah Pirikahu^1^; Jane Walter^2^; Gemma Cadby^1^; Ellie Darcey^1^; Rachel Lloyd^1^; Martha Hickey^3^; Christobel Saunders^4^; Michael Hackmann^5,6^; David D Sampson^7^; John Shepherd^8^; Lothar Lilge^2,9^; Jennifer Stone^1^

Corresponding author: Jennifer Stone, Genetic Epidemiology Group, School of Population and Global Health, 35 Stirling Highway M431, The University of Western Australia, Crawley, Western Australia, 6009 Australia. [Jennifer.stone@uwa.edu.au](mailto:Jennifer.stone@uwa.edu.au) ORCID: 0000-0001-5077-0124

**Supplementary figure 1.** The optical breast spectroscopy device used in this study to measure breast tissue components %water and %collagen. **A**. Prototype of the OBS device holding on to the breast during an OBS scan. **B**. Inside of a sample breast cup displaying photodetectors^24^.

**
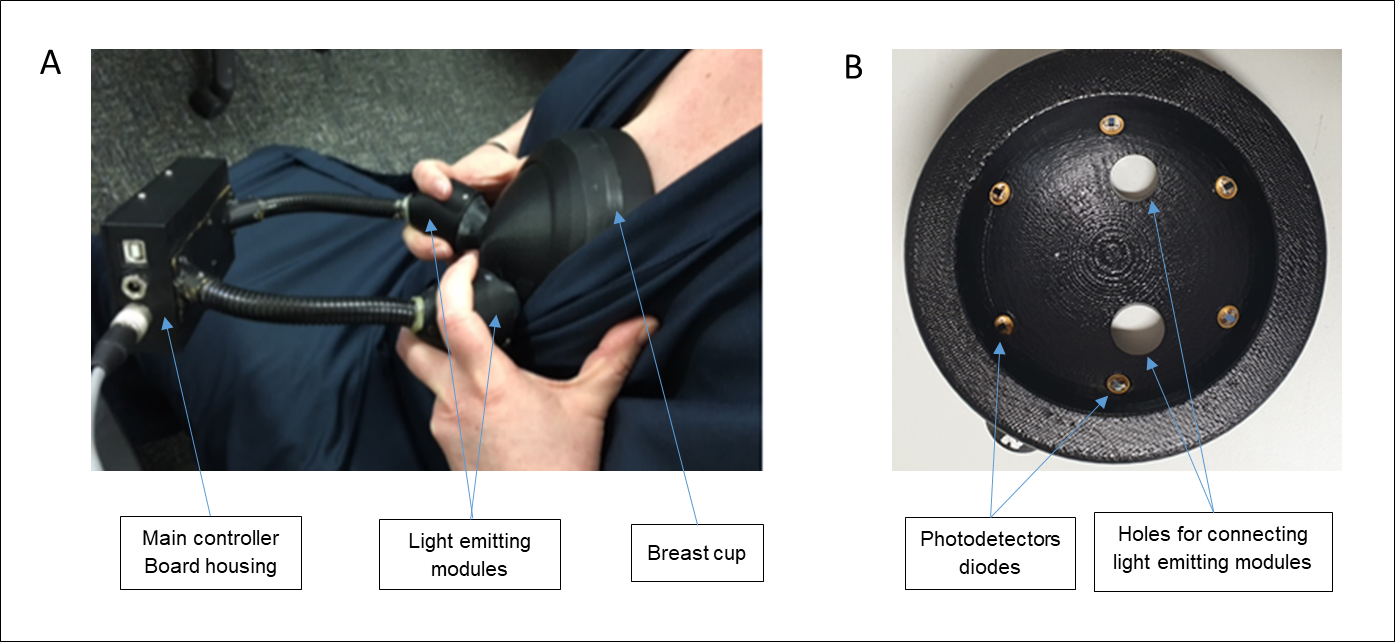
**

**Supplementary Figure 2.** Breast density distribution in younger women (18-40) and older women (>40) in the study. **a**. OBS-%water+%collagen distribution in younger women and older women. **b**. DXA-%FGV distribution in younger women and older women.


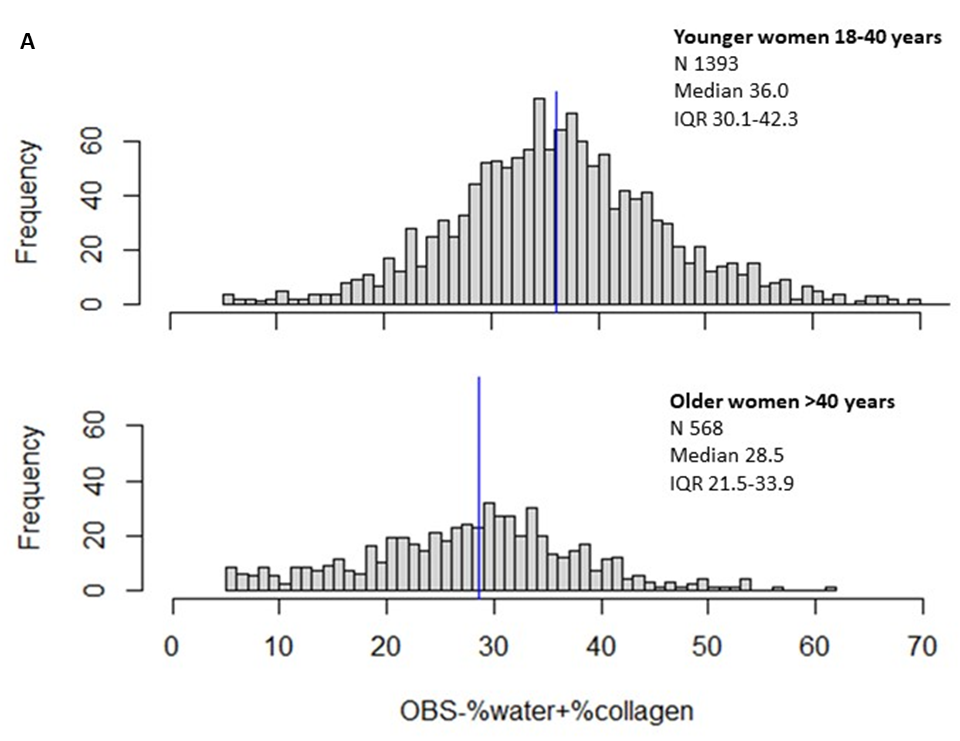


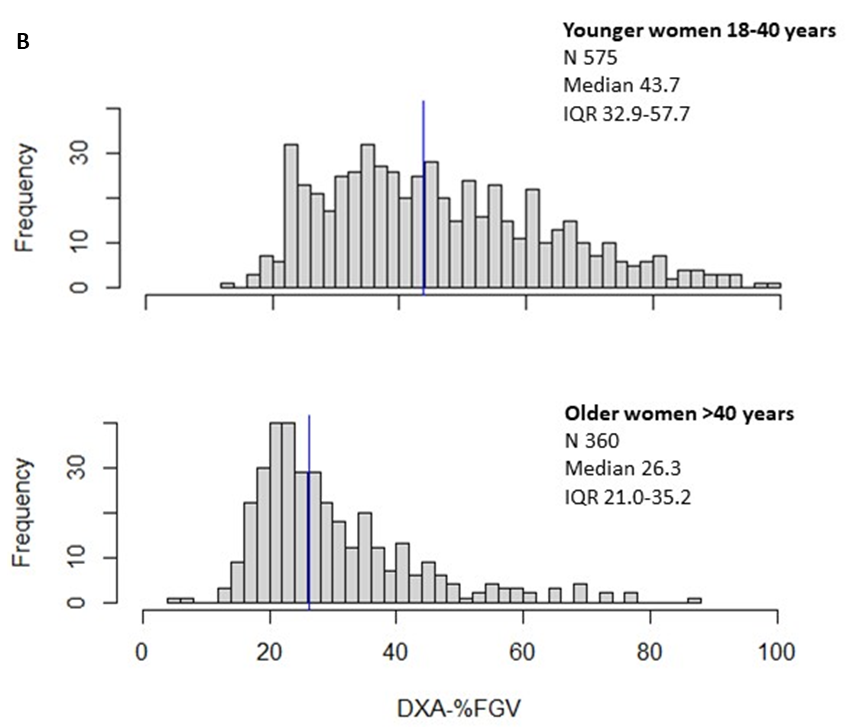


**Supplementary** F**igure 3.** The distribution of BMI, stratified by age category for all the participants with an OBS measurement. Within each age category, all points are plotted, along with a histogram and a boxplot. The number labelled on the histogram is the median of the distribution.


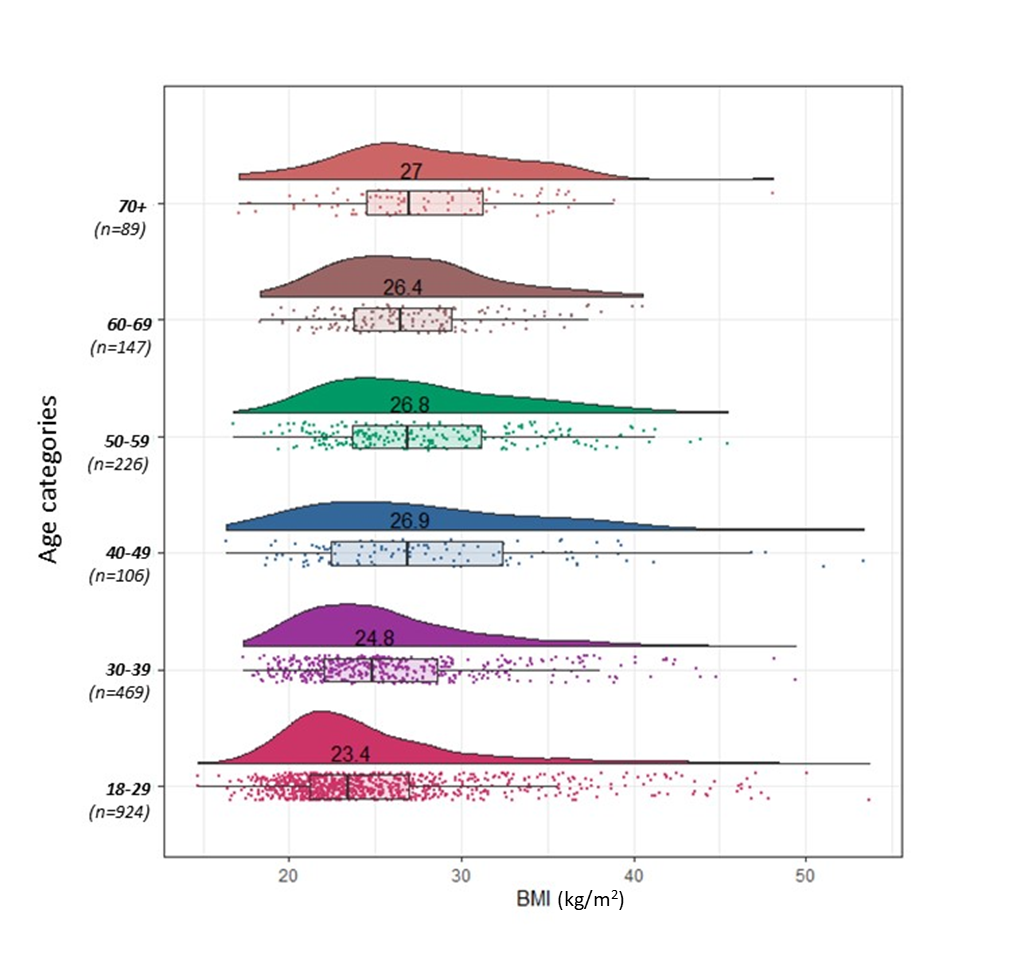

Supplement: Supplementary file 1 — Supplementary file1 (DOCX 2102 kb) [file 10549_2024_7269_MOESM1_ESM.docx]
